# Meeting report: the 2021 FSHD International Research Congress

**DOI:** 10.1186/s13395-022-00287-8

**Published:** 2022-01-17

**Authors:** Sujatha Jagannathan, Jessica C. de Greef, Lawrence J. Hayward, Kyoko Yokomori, Davide Gabellini, Karlien Mul, Sabrina Sacconi, Jamshid Arjomand, June Kinoshita, Scott Q. Harper

**Affiliations:** 1 Department of Biochemistry and Molecular Genetics, University of Colorado Anschutz Medical Campus, Aurora, CO 80045 USA; 2 Department of Human Genetics, Leiden University Medical Center, Leiden, The Netherlands; 3 Department of Neurology and Wellstone Center for FSHD, University of Massachusetts Medical School, Worcester, MA 01655 USA; 4 Department of Biological Chemistry, School of Medicine, University of California, Irvine, CA 92697 USA; 5 Division of Genetics and Cell Biology, IRCCS San Raffaele Scientific Institute, Milan, Italy; 6 Department of Neurology, Donders Institute for Brain, Cognition, and Behaviour, Radboud University Medical Center, Nijmegen, The Netherlands; 7 Nice University Hospital/Institute of Research on Cancer and Aging of Nice, Nice, France; 8 FSHD Society, Randolph, MA USA; 9 Center for Gene Therapy, The Abigail Wexner Research Institute at Nationwide Children’s Hospital, Department of Pediatrics, The Ohio State University College of Medicine, Columbus, OH 43205 USA

## Abstract

Facioscapulohumeral muscular dystrophy (FSHD) is the second most common genetic myopathy, characterized by slowly progressing and highly heterogeneous muscle wasting with a typical onset in the late teens/early adulthood [1]. Although the etiology of the disease for both FSHD type 1 and type 2 has been attributed to gain-of-toxic function stemming from aberrant DUX4 expression, the exact pathogenic mechanisms involved in muscle wasting have yet to be elucidated [2–4]. The 2021 FSHD International Research Congress, held virtually on June 24–25, convened over 350 researchers and clinicians to share the most recent advances in the understanding of the disease mechanism, discuss the proliferation of interventional strategies and refinement of clinical outcome measures, including results from the ReDUX4 trial, a phase 2b clinical trial of losmapimod in FSHD [NCT04003974].

## Keynote presentations

Each day of the Congress began with a keynote presentation, followed by dedicated sessions on specific areas of interest. On day 1, Russell Butterfield (University of Utah) presented “The history of FSHD in a large Utah kindred: The fruits of 80+ years of engagement,” a retrospective of the clinical manifestation of > 2000 FSHD descendants of a gene carrier who emigrated to Utah in the nineteenth century [5, 6]. This kindred represents a unique resource to identify genetic modifiers for FSHD and to develop treatments that target key pathways identified by these genetic modifiers. Research performed thus far has shown that all affected family members carry a 20-kb (6 unit) D4Z4 repeat array, confirming the meiotic stability of the founder mutation [5]. In 2018, Drs. Butterfield and Weiss (both at the University of Utah), in collaboration with Charles Emerson and the University of Massachusetts Wellstone Muscular Dystrophy Cooperative Research Center, began a genetic modifier study to test the hypothesis that the clinical severity of the founder mutation could be modified by common genetic variants. For this study, a collection of 566 DNA samples from Kindred 1462 (K-1462), for which the founder mutation had been segregating for at least 170 years and clinical data collected for over 80 years, has been assembled. Preliminary whole-genome sequencing of 4 individuals from K-1462 identified 4 rare single nucleotide polymorphisms (SNPs) associated with the risk haplotype, but a higher resolution method would be needed to further characterize the phenotype(s). For this, a targeted enrichment approach using Nanopore single-molecule sequencing was set up (discussed in more detail by Quentin Gouil in session 2). Future steps include a genome-wide association study using DNA microarrays to study unlinked modifiers and Nanopore sequencing to study the linked and unlinked modifiers. Recruitment of additional family members from the kindred is ongoing.

The second keynote presentation was given by Stephen Tapscott (Fred Hutch Cancer Research Center) and explored the aspects of immune response in FSHD. Several previous studies have suggested an involvement of the complement system in FSHD, including the presence of an activated membrane attack complex in the FSHD muscle and sarcolemmal composite deposits of C5b-9. RNAs encoding complement proteins are mildly upregulated during FSHD progression, and the secretome of DUX4-expressing cells in culture shows the presence of complement proteins. Exploration of complement as an FSHD biomarker showed that C3 and C4b were significantly elevated in FSHD populations, while a composite score including a panel of four complement proteins showed promise as an FSHD biomarker and supported a role for complement activation in FSHD. Evidence from a pulsatile DUX4 expression system points to a model where the expression of immunogenic proteins by DUX4 and its suppression of MHC class I antigen presentation cause a fluctuating immune response that could ultimately contribute to FSHD pathology.

## Session 1: Discovery research

Studies show that even brief exposure to DUX4 may trigger self-sustaining molecular and pathological cascades relevant to toxicity in FSHD muscle [7]. Much remains to be learned about endogenous regulators of DUX4 with the potential to be manipulated for therapeutic benefit, and rapid ongoing advances in single-cell/nucleus transcriptome analysis are elucidating important effects of DUX4 in specific cell types within the native milieu of FSHD muscle.

Although DUX4 is widely thought to be the key trigger for FSHD, the protein has been difficult to detect in adult muscle biopsy tissue, raising the possibility that DUX4 might act only transiently. Darko Bosnakovski (University of Minnesota) studied a mouse model (iDUX4pA;HSA) in which DUX4 expression can be induced using doxycycline (Dox) and examined how transient DUX4 expression affects downstream target genes, inflammation, and muscle regeneration [8]. Muscle pathology resembling human FSHD and transcriptomic changes consistent with those from MRI-guided FSHD muscle biopsies was observed in the mice continuously induced with Dox for 4 months although DUX4 protein was detectable only rarely in a small proportion of myofibers. Increased fibroadipogenic progenitors (FAPs) expressing a profibrotic transcriptome signature were detected in the affected muscles. Treatment of mice with an anti-fibrotic drug led to less inflammatory infiltration and intramuscular collagen deposition. Whereas a single intraperitoneal injection of Dox induced DUX4 expression within a day, DUX4 decayed to almost undetectable levels 4 days after the pulse. At that time, infiltrates of mononuclear cells had appeared, and markers of inflammation and mouse biomarkers of DUX4 activity peaked; importantly, the latter were still detected at least 8 days after induction. Increased FAPs persisted for at least 30 days after a single DUX4 burst, and muscles injured with cardiotoxin showed impaired regeneration. Furthermore, 3 months after transient induction of DUX4 with Dox chow for 10 days, FAPs were still elevated, and the muscle fibers switched to a slow oxidative fiber type. Taken together, these findings are consistent with the observations from human muscle biopsies with active FSHD and support the view that even transient DUX4 expression can provoke long-term pathological effects.

DUX4 regulation and mechanisms of toxicity likely are influenced by other cellular components that could be relevant to therapeutic development for FSHD. Paola Ghezzi (San Raffaele Scientific Institute, Milan) identified ten DUX4-interacting proteins using double affinity purification at high stringency and a proteomic approach. Apoptosis of transfected cells expressing DUX4 increased upon knockdown of one of these interactors, matrin 3 (MATR3), a nuclear matrix protein involved in gene expression, RNA control, and the DNA damage response. It is noteworthy that mutant forms of MATR3 have been associated with ALS and a distal myopathy. Furthermore, co-transfection with a MATR3 construct specifically relieved DUX4-mediated apoptosis. Fragment expression of MATR3 showed that amino acids 1–287 were sufficient to protect from DUX4-induced apoptosis, and MATR3 interacted directly with the DNA-binding domain of DUX4. A proximity ligation assay showed that MATR3 and DUX4 interact in FSHD primary muscle cells, and overexpression of MATR3 in these cells decreased DUX4 and its target gene expression, while the converse occurred upon siRNA-mediated depletion of MATR3. Likewise, MATR3 overexpression improved the myogenic differentiation, fusion index, and viability of FSHD muscle cells while its depletion increased apoptosis. A preliminary working model was presented in which MATR3 interaction with DUX4 may limit DUX4 availability to bind to DNA and initiate target gene transcription. This study paves the way for further structural analysis of the MATR3-DUX4 interaction that may allow the design of future drug-like agents with similar properties.

Treatments modulating DUX4 expression target the root cause of FSHD, but downstream consequences of DUX4 that affect the muscle microenvironment are not fully understood. To help define these changes, Anugraha Raman (Fulcrum Therapeutics) presented the results of single-nucleus RNA sequencing from 16 FSHD STIR-positive and two healthy muscle biopsy samples. These yielded ~ 126,000 disease nuclei and ~ 23,000 healthy nuclei which could be assigned to diverse known cell types based on marker gene expression from previous muscle transcriptomics studies. Myonuclei comprised ~ 60% of the cell types present (decreased from ~ 90% in healthy biopsies), and cell types previously identified in pathological samples were increased in number, including fibroadipogenic progenitors or FAPs (increased to 13%), adipocytes, T and B cells, and macrophages. These changes in cell type composition were highly variable among individual biopsies, reflecting the expected variability in clinical severity and sampling from small biopsies. Moreover, gene expression changes in specific cell types could be linked to FSHD biopsy signatures from bulk RNAseq analysis, which previously identified ~ 170 dysregulated genes. This may suggest how particular cell types contribute to these FSHD signatures. Furthermore, DUX4 target gene expression was limited to a subset of 30 rare myonuclei within a cluster of cells linked to regeneration or early myogenesis, amounting to only 1 in ~ 3000 total myonuclei. Ongoing analysis of these results may provide insights regarding microenvironmental changes during FSHD that could suggest new therapeutic targets, as well as the utility of biopsies to track DUX4 target genes to serve as biomarkers during clinical trials.

The highlights of the poster session included a detailed biochemical analysis by Alexandra D. Gurzau (University of Melbourne) of the dimerization of *Structural Maintenance Of Chromosomes Flexible Hinge Domain Containing 1* (*SMCHD1*)’s GHKL ATPase, which requires its N-terminal ubiquitin-like domain and appears critical for chromatin interaction and gene silencing by SMCHD1. Jonathan Chau (University of California, Irvine) observed that most endogenous *DUX4* transcripts accumulate and are retained in the foci within the muscle cell nucleus and that some DUX4 target genes such as *LEUTX* can sustain downstream changes once triggered by DUX4. Rajanikanth Vangipurapu (Saint Louis University) used CRISPR methodology to knockout p38α/β MAP kinases individually and in combination and showed that p38 appears to be an important, although not exclusive, activator of DUX4 expression in FSHD cells. Chris Brennan (Pfizer Rare Disease Research) measured global changes in protein phosphorylation after DUX4 induction and showed that DUX4 overexpression for 14 h leads to changes in > 600 mRNA splice variants.

## Session 2: Genetics and epigenetics

Emanuele Mocciaro (San Raffaele Scientific Institute) presented a novel druggable target to repress pathological *DUX4* expression as a possible FSHD therapy. Previously, their laboratory discovered that the long non-coding RNA termed *DBE-T* is transcribed upstream of the *DUX4* gene and effectively stimulates *DUX4* expression in FSHD patient cells [9]. *DBE-T* recruits trithorax group protein ASH1L to the D4Z4 repeat region to mediate *DUX4* activation, though the exact molecular mechanism is unknown. To address how *DBE-T* functions, they set up a reporter system to first identify the minimum domain of *DBE-T* RNA necessary and sufficient for the gene activating function of *DBE-T*. Using this fragment (fragment 3) as bait, the proteomic study identified WD40 repeat-containing protein 5 (WDR5) as one of the interacting proteins. Overexpression and depletion of WDR5 against *DBE-T* with and without the fragment 3 confirmed that WDR5 is essential for *DBE-T* (fragment 3)-mediated gene activation. In FSHD cells, depletion or chemical inhibition of WDR5 effectively suppressed *DUX4* and DUX4-target gene expression and rescued the DUX4 overexpression phenotype to the comparable extent as DUX4 depletion. WDR5 has multiple functions in chromatin-mediated gene regulation and is best known as a core component of the mixed-lineage leukemia (MLL) histone H3K4 methyltransferase complex together with ASH2L. The current study by Mocciaro et al. suggests that ASH1L may form a similar complex. WDR5 is known to bind to enhancer-like long non-coding RNAs (lncRNAs), including HOTTIP in the HOXA locus, to mediate gene activation. Thus, *DBE-T* appears to play a similar role through interaction with WDR5 to activate *DUX4*. They are currently testing the efficacy of WDR5 inhibitors in FSHD cell and animal models. While the side effects are of concern considering the complex roles of WDR5, the inhibitors have been tested for cancer treatment and, thus, may be amenable to repurposing for FSHD treatment.

Quentin Gouil (Walter and Eliza Hall Institute of Medical Research) presented their study to use nanopore long-read sequencing to streamline FSHD genetic and epigenetic diagnostics in a more affordable manner. Nanopore sequencing is superior to other sequencing platforms in terms of the length and accuracy of the sequencing reads. Gouil presented examples to demonstrate that it is possible to accurately determine the haplotype and allele-specific D4Z4 repeat lengths as well as large structural rearrangements involving D4Z4 alleles. In addition, their data indicated that it is possible to detect a single-nucleotide change in the long *SMCHD1* gene locus and to analyze DNA methylation status at 4q35. Gouil also presented the results of the targeted nanopore sequencing using a Cas9-based tagging approach, which is more cost-effective. This strategy can also be used for multiplexing so that sequencing of D4Z4 arrays and modifier genes can be performed at the same time with high enrichment and accuracy. They hope to further refine the technology to combine other epigenetic changes such as chromatin opening and histone modifications.

## Session 3: Pathology and disease mechanisms

To develop targeted therapies for FSHD, a detailed understanding of the regulatory pathways for DUX4 expression, the downstream cascades instigated by DUX4, and extra-muscular cells contributing to disease pathophysiology would be important. In this session, novel cellular models of the disease, insight on DUX4 regulation at the protein level, and a potential mechanism for muscle inflammation were presented.

In about 4% of FSHD patients, the disease is associated with mutations in the *SMCHD1* gene [3]. Curiously, mutations in the same gene also cause Bosma arhinia microphthalmia syndrome (BAMS) [10]. Camille Laberthonniѐre (Marseille Medical Genetics) used induced pluripotent stem cells (iPSCs) to generate muscle fibers from FSHD and BAMS-affected individuals to conduct an in-depth transcriptomics study. Using a new computational framework, MOGAMUN, they could identify pathways that were common to or distinct between the two diseases. While the FSHD muscle fibers showed an effect on skeletal muscle function, the BAMS muscle showed a more pronounced effect on developmental processes. More specifically, the FSHD muscle highly downregulated sarcomere components, including several actin and myosin filament proteins, as well as proteins involved in calcium handling. Functional studies validated sarcomeric defects in FSHD iPSC-derived muscle fibers, uncovering a link between muscle weakening in FSHD to alteration of the contractile apparatus.

In recent years, we have learned significant information regarding the regulation of *DUX4* at the RNA level, but little about DUX4 protein regulation. Renatta Knox (Nationwide Children’s Hospital) presented work investigating post-translational modifications (PTMs) of the DUX4 protein, their consequence for DUX4 function, and whether such PTMs can be targeted as a therapeutic approach. Using mass spectrometry, Knox and colleagues in the Harper lab discovered several serine and threonine phosphorylation sites and arginine methylation sites on DUX4. Meticulous screening of 55 different mutants that either mimic or ablate such modifications led to the identification of a subset of serine/threonine phosphomimetic mutants and an arginine methylation null mutant which protected cells against DUX4-mediated toxicity. In follow-up studies, they demonstrated that DUX4 is a substrate of Protein Kinase A and that it also exists in complex with the arginine methyltransferase PRMT1. Both broad methylation inhibitors and specific PRMT1 inhibitors could protect against DUX4-induced cell death in myoblasts, demonstrating the therapeutic promise of targeting DUX4 PTMs.

To eliminate the variability associated with using samples isolated from different donors, Nam Viet Nguyen (University of California, Irvine) used CRISPR-Cas9 to generate isogenic FSHD models by inducing deletions of D4Z4 repeats or mutations in *SMCHD1* starting from the same immortalized muscle cell line from a healthy donor. By deleting D4Z4 repeat units and the *SMCHD1* gene individually or in combination, Nguyen was able to recapture the molecular characteristics of FSHD including reduced H3K9me3 at the D4Z4 locus and expression of DUX4 targets. DUX4 target gene expression was particularly robust in the double mutants and revealed interesting temporal patterns regarding early and late targets. Interestingly, the mutant cells differed from patient-derived myoblasts in terms of the timing of DUX4 expression compared to DUX4 target expression, with the mutant cells showing activation of targets only after differentiation into myotubes. These results hint at interesting regulatory mechanisms that can be explored using these novel FSHD model cell lines.

One of the prominent features of FSHD and of *DUX4*-based models is muscle inflammation. To investigate the mechanism underlying this process, Anna Greco (Radboud University Medical Center) studied the circulating markers from 150 FSHD patients and 98 healthy controls and found IL-6 and TNF-α to be significantly upregulated in FSHD, with IL-6 positively correlating with muscle weakness, disease severity, and duration. Working ex vivo, she found similar results after stimulation of whole skeletal muscle or purified natural killer (NK) cells. Based on these results, she proposed a model where muscle cells from FSHD patients produce inflammatory molecules, which then activate NK cells in the circulation, to further produce inflammatory cytokines and contribute to the disease. If correct, interfering with this process might slow down disease progression in FSHD.

## Session 4: Biomarkers

Within the FSHD field, there is a high need for molecular biomarkers that correlate with disease severity and progression, and that can be used for the evaluation of treatment strategies. In this session, several laboratories presented their work on the identification of FSHD biomarkers.

Amy Campbell (University of Colorado Anschutz Medical Campus) used the Proximity Extension Assay from Olink with the goal to identify DUX4-driven genes that can be detected in the blood [11]. This assay uses matched pairs of DNA-coupled antibodies that can bind the biomarker of interest followed by quantitative PCR to quantify protein levels. The Olink panels contain a limited number of DUX4 target gene peptides consisting of alkaline phosphatase, placental (ALPP), carbonic anhydrase 2 (CA2), and corticotropin-releasing hormone-binding protein (CRHBP). The expression of these candidate biomarkers were studied in doxycycline-inducible DUX4 (MB35-iDUX4) and patient-derived myoblasts. While CRHBP and CA2 levels were below the limit of detection, ALPP levels could be readily detected in cell lysates and supernatants from DUX4-expressing cells. *DUX4* siRNA treatment of MB35-iDUX4 myoblasts resulted in ALPP supernatant levels that were below the limit of detection. By studying myoblast cell lines from control individuals and patients with FSHD at distinct days of differentiation, an increase in ALPP levels in supernatants of FSHD cell lines was confirmed. Experiments with small molecular inhibitors of DUX4 (BET1 inhibitor JQ1, β2 agonist formoterol, p38 inhibitor losmapimod) next showed a reduction in ALPP supernatant levels which corresponded with reduced DUX4 expression levels. ALPP serum levels in 20 individuals with FSHD were however unchanged compared to ALPP serum levels in 20 control individuals. One explanation may be that ALPP basal levels were already quite high, suggesting that non-muscle expression of ALPP may mask the muscle-specific differences. Taken together, ALPP does not seem to be a promising FSHD serum biomarker, but ALPP supernatant levels can be used for tracking DUX4 activity in cultured cells.

Robert Bloch (University of Maryland School of Medicine) presented ongoing work of his group on the potential FSHD biomarker solute carrier family 34 member 2 (SLC34A2), a protein responsible for sodium-dependent phosphate uptake into cells and a DUX4 target gene. Previous research from this laboratory using human muscle xenografts of immortalized FSHD and control myogenic precursor cells identified SLC34A2 as a novel protein biomarker for FSHD [12]. Furthermore, western blot analysis showed that SLC34A2 protein can be detected in FSHD myoblasts and FSHD xenografts and is increased in the serum from mice with FSHD xenografts compared to the serum from mice carrying control xenografts. In addition, in 10 patients with FSHD SLC34A2, protein levels were 3–9 times higher compared to SLC34A2 serum levels in a control individual. Collectively, these data show that SLC34A2 may be a useful serum protein biomarker for FSHD. Future studies include measurements of SLC34A2 protein levels in additional control sera.

Jonathan Pini (Université Côte d’Azur) discussed a retrospective study in serum samples from 100 genetically confirmed adult patients with FSHD by measuring the levels of 20 pro-inflammatory and regulatory cytokines using Meso Scale Discovery kits. In addition, clinical data was available for this patient cohort, including Manual Muscle Testing Sum Scores (analysis of 28 muscles from upper and lower limbs), Brooke and Vignos scores (functional assessment of upper and lower limbs), and clinical severity scores. Only the concentrations of the cytokines IL-6 and TNF-α correlated with functional and clinical severity scores, and the correlation was the highest for IL-6. In addition, IL-6 serum levels were 2–3 times higher in patients with FSHD1 than in 50 matched control individuals. Finally, IL-6 serum levels were significantly higher in FSHD1 patients with a clinical severity score between 7 and 10. Studies in a DUX4-inducible transgenic mouse model, the ACTA1-MCM;FLExDUX4 mouse model, next showed that both serum and muscle IL-6 levels were significantly increased in mice with the highest DUX4 expression levels and a severe phenotype [13]. In conclusion, IL-6 shows promise as a serum biomarker for FSHD that correlates with disease severity. This study is now published [14]. In addition, IL-6 may be a therapeutic target, and IL-6 inhibitors have already been approved for the treatment of several disorders, including for rheumatoid arthritis.

## Session 5: Interventional strategies

Given its importance for FSHD, interfering with DUX4 expression or function has strong therapeutic relevance, and this session highlighted the strategies for blocking DUX4 transcription or inducing *DUX4* RNA degradation.

Fran Sverdrup (Saint Louis University) characterized the role of p38 mitogen-activated protein kinase in the regulation of *DUX4* transcription and reported that p38 α/β have a key role in the activation of *DUX4* expression during differentiation of FSHD muscle cells. Treatment with the p38 inhibitor losmapimod significantly reduced DUX4 expression and activation of DUX4 target genes. Using a mouse xenograft model, the Sverdrup group found that losmapimod was highly effective at reducing the peak of DUX4 activation during early stages of xenograft development but that low levels of DUX4 target genes persisted that were insensitive to p38 inhibition at later stages. The relevance of these findings remains to be determined. These results on the mechanism of action of losmapimod are important to the ongoing phase 2b clinical trial in FSHD (see the “Special session—Fulcrum’s phase 2b ReDUX4 results” section).

An alternative way to downregulate DUX4 expression is by degrading its RNA. Using in silico predictions, Nizar Saad (Nationwide Children’s Hospital) found eight potential *miR-675* binding sites in the *DUX4* mRNA, with two high-affinity *miR-675* binding sites being confirmed by in vitro binding assays. After optimization of the *miR-675* flanking sequences, a construct significantly reducing DUX4 expression and DUX4-associated toxicity in HEK293 cells ectopically expressing DUX4 was identified. Intriguingly, blocking endogenous *miR-675* activity increased the expression of DUX4 and one of its targets in FSHD muscle cells, confirming the idea that *miR-675* is a natural regulator of *DUX4* expression. Using an AAV-based in vivo model, treatment with *miR-675* caused a significant reduction of DUX4 expression and of the associated pathological signs. Notably, compounds increasing *miR-675* expression and leading to a concomitant decrease in DUX4 levels were identified. Future work in animal models will investigate the therapeutic relevance of *miR-675* for FSHD [15].

## Session 6: Antisense strategies

Several groups in academia and industry are pursuing nucleic acid-based strategies to inhibit *DUX4* gene expression at the RNA level. Although there were some differences among the various strategies, particularly in the mechanism of action and delivery strategy, each shared a common feature: the requirement for the therapeutic nucleic acid species to form antisense base pairs with the *DUX4* mRNA. The speakers in this session described 3 different mechanisms to accomplish *DUX4* mRNA silencing with antisense approaches:

1. RNA interference (RNAi) strategies utilizing small interfering RNAs (siRNAs) designed to bind *DUX4* mRNA and trigger its degradation by the RNA-induced silencing complex (RISC) (Barbora Malecova, Avidity Biosciences; Katelyn Daman, University of Massachusetts; Jonathan Van Dyke, Arrowhead; Lindsay Wallace, Nationwide Children’s Hospital)
2. Induction of RNAse H cleavage via RNA/DNA duplex using DNA gapmer antisense oligonucleotides (gapmer ASOs, or gapmers) (Linde Bouwman from Leiden University Medical Center)
3. Steric hindrance using phosphorodiamidate morpholino oligomers (PMOs or Morpholinos) (Ngoc Lu-Nguyen, Royal Holloway University, and Nelson Hsia, Dyne)

Historically, the delivery of synthetic nucleic acids to the muscle has been challenging [16]. The presenters in this session described various methods to increase nucleic acid delivery to the muscle, including formulating nucleic acids with lipids (Linde Bouwman, Leiden University Medical Center; Katelyn Daman, University of Massachusetts), cell-penetrating dendrimers (Ngoc Lu-Nguyen, Royal Holloway University), or linking with antibodies or ligands targeting the Transferrin receptor (TfR) or other receptors present on muscle membranes (Barbora Malecova, Avidity Biosciences; Nelson Hsia, Dyne; Jonathan Van Dyke, Arrowhead). Like other traditional small molecule therapies, those employing ASOs and siRNAs require repeated lifelong administration to maintain an intended therapeutic effect. Alternatively, AAV-based muscle gene therapy systems use viral capsids to achieve widespread muscle delivery and can be designed to enable long-term expression following one administration (Lindsay Wallace, Nationwide Children’s Hospital). Current limitations of AAV vectors for muscle gene therapy include high production costs, the inability to adjust dose and readminister once a vector is delivered, and safety concerns associated with systemic delivery of high vector doses.

Linde Bouwman (Leiden University Medical Center) reported results from a study using uninduced ACTA1-MCM;FLExDUX4 mice, where DNA gapmers, mixed with Palmitate (C16), were delivered twice a week at 50 mg/kg for 4 weeks, followed by 50 mg/kg weekly for another 5 weeks. Compared to ACTA1-MCM;FLExDUX4 mice treated with a control gapmer, DUX4-gapmer-treated animals showed reductions in *DUX4* and 3 DUX4-activated mouse target genes, and evidence of histological and functional improvement (reduction in myofibers with central nuclei from 26 to 18%, and 30% increase in 1250 meter treadmill performance, respectively). However, no improvements were found in muscle weight or strength measured using a hanging grid test and a grip strength test. This study is now published [17].

Barbora Malecova (Avidity Biosciences) presented in vitro and in vivo data using DUX4 target genes as a surrogate indicator of silencing efficacy, following treatment with *DUX4*-targeting siRNA conjugated to a murine Transferrin receptor (TfR) monoclonal antibody. Specifically, Dr. Malecova reported reductions in 4 DUX4 target genes (QPCR) and SLC34A2 protein (immunofluorescence) in 11 different human FSHD myotube lines treated with 10 nM siRNA. Similarly, dose-dependent reductions in 4 DUX4-activated mouse target genes were found in FLExDUX4 mice treated with siRNA+TfR antibody conjugate, 3 weeks later. No functional or histological outcomes were reported for the mouse study (Table 1).

**Table 1:** Silencing mechanism, nucleic acid species, and muscle delivery systems described in session 6 of the FSHD IRC meeting. ^a^Ref 19. ^b^Ref 20. ^c^Ref 18. ^d^Used FM10, a previously published sequence targeting the *DUX4* polyA signal. Although the PMO operates via steric hindrance, blocking polyadenylation could lead to *DUX4* mRNA instability and degradation (Ref 18)

Speaker, affiliation	DUX4 silencing mechanism	Antisense species	Conjugate/muscle delivery system
Katelyn Daman, University of Massachusetts	RNAi	siRNA	Docosanoic acid
Barbora Malecova, Avidity Biosciences	RNAi	siRNA	Murine TfR mAb
Jonathan Van Dyke, Arrowhead	RNAi	siRNA	Not specified
Lindsay Wallace, Nationwide Children’s Hospital^a,b^	RNAi	In vivo expressed microRNA	AAV6 and AAV9 gene therapy vectors
Linde Bouwman, Leiden University Medical Center	RNAse H	Gapmer ASO	Palmitate (C16)
Ngoc Lu-Nguyen, Royal Holloway University^c^	Steric hindrance	PMO	Vivo-PMO, cell penetrating guanidinium dendrimer
Nelson Hsia, Dyne	Steric hindrance^d^	PMO	TfR antibody Fab fragment

Katelyn Daman (University of Massachusetts) used *DUX4*-targeting siRNAs formulated with docosanoic acid to facilitate delivery to FSHD myoblasts, FSHD myotubes, and an FSHD xenograft model. In vitro treatment of FSHD cells with 3 different concentrations of siRNA (0.5, 1, and 2 μM) produced dose-dependent reductions in 3 human DUX4 target transcripts. Similarly, 4 DUX4 target genes were reduced in FSHD xenograft mice treated subcutaneously with two 20 mg/kg doses of siRNA/docosanoic acid over a week.

Nelson Hsia (Dyne Therapeutics) described a strategy to deliver the *DUX4*-targeting PMO FM10 to FSHD myotubes by coupling the therapeutic oligonucleotide to a TfR antibody Fab fragment. FM10 had been previously reported to bind atop the *DUX4* polyA signal, thereby potentially operating to destabilize the *DUX4* mRNA by blocking polyadenylation [18]. Dr. Hsia reported reductions in 3 DUX4 target genes in human FSHD myotubes treated with 8 nM of the FM10-TfR antibody Fab fragment conjugate.

Ngoc Lu-Nguyen (Royal Holloway University) presented her work describing an in vitro screen of several PMOs targeting *DUX4*, followed by coupling of a lead sequence to a guanidinium dendrimer for in vivo delivery (Vivo-PMO). In vitro, several PMOs caused reductions in DUX4 and 3 target genes in FSHD myotubes. In vivo studies were performed in tamoxifen-induced ACTA1-MCM;FLExDUX4 mice, where animals received weekly intraperitoneal doses (10 mg/kg) of the lead Vivo-PMO for 30 days. This treatment led to partial reductions in *DUX4* and 2 DUX4-activated mouse target genes and partial improvement in histological and functional outcomes. This study is now published [19].

Jonathan Van Dyke (Arrowhead Pharmaceuticals) presented a comprehensive study of *DUX4* inhibition involving the delivery of a nucleic acid with an unspecified delivery system. The data reported suggested that the siRNAs and delivery system were highly effective at silencing *DUX4* and improving several phenotypes. Specifically, in vitro delivery of 1, 10, and 100 nM *DUX4*-targeting siRNAs to human FSHD myotubes caused dose-responsive reductions in 10 DUX4 target genes. For in vivo studies, Arrowhead used the tamoxifen-induced ACTA1-MCM;FLExDUX4 model. Animals were treated with conjugated siRNA on days 3 and 5 after tamoxifen induction, followed by weekly injections thereafter for up to 30 days. This treatment regimen caused reductions in *DUX4* and a DUX4-activated mouse target gene, improvement in body weight at 30 days, and increased performance on the rotarod 22 days after DUX4 induction. Improvements in fibrosis were also reported but not quantitated.

Lindsay Wallace (Nationwide Children’s Hospital) reported her progress on translating an AAV-based gene therapy project aimed at inhibiting *DUX4* with an engineered microRNA called *mi405* [20, 21]. Dr. Wallace reported several in vivo studies in both the uninduced and tamoxifen-induced TIC-DUX4 mouse models, which recapitulate mild and more severe forms of DUX4-related myopathy, respectively [22]. To address the durability of treatment, a single intramuscular dose of 1 × 10^11^ vector genomes (vg) of AAV6 serotype vectors carrying a U6.mi405 expression cassette protected muscles from histological damage out to 1 year (25% central nuclei in untreated limbs; 5% in treated limbs), and systemic intravenous (IV) delivery of 3 × 10^13^ or 3 × 10^14^ vg/kg improved activity, hindlimb rearing and rotarod performance out to 6 months. Similarly, tamoxifen-induced TIC-DUX4 mice treated IV with AAV6 or AAV9 serotyped U6.mi405 vectors showed no decline in cage activity over a 10-week period. Finally, in a collaborative study with the Emerson lab (University of Massachusetts) using an FSHD xenograft model, IM injection of AAV6 vectors reduced *DUX4* and 8 DUX4 target genes.

Finally, Dr. Yi-Wen Chen, who presented an ASO study at the 2020 IRC meeting that is now published, did not present an abstract this year but participated in the Q&A at the end of this session [23].

## Session 7: Clinical studies and outcome measures

With ongoing and various upcoming clinical trials on targeted therapies in FSHD, the main focus of this session was on clinical trial preparedness.

The first presentation by Sanne Vincenten (Radboud University Medical Center) was on the preliminary muscle MRI results of a 5-year natural history study in FSHD. Because clinical outcome measures might not be sensitive enough to detect a change in this slowly progressive disease, quantitative muscle MRI has been proposed as a surrogate measure or biomarker for FSHD clinical trials. This study included 105 FSHD patients and determined the fat fraction (FF) of 19 leg muscles per patient at baseline and 5-year follow-up. There was a significant progression of FF of almost all muscles, most prominent in the hamstring and calf muscles. The average FF of all muscles combined (MRI compound score) increased by 2.4 (± 2.8 SD). Longitudinal correlations between the change in MRI compound score and the change in two clinical outcome measures (Lamperti clinical severity score and Motor Function Measure) were moderate but significant (CC 0.3). The change in MRI compound score did not correlate to FSHD type, sex, D4Z4 repeat array size, or age (CC 0.0-0.2). The change in MRI compound score correlated moderately to the baseline Lamperti clinical severity score, and number of STIR-positive muscles and degree of fatty infiltration at baseline (CC 0.3–0.4, *p* < 0.05). These preliminary results show a relation between changes in quantitative MRI and clinical outcome measures, but additional work is ongoing to determine the optimal use of MRI in FSHD clinical trials.

Next, Ghobad Maleki and Ahnjili Zhuparris (Centre for Human Drug Research) presented a study on the feasibility of using smartphones and wearables to capture FSHD-related symptoms and overcome the limited ability of the existing clinical outcomes by continuous monitoring changes relevant to the disease. Thirty-eight FSHD patients and 20 non-FSHD control subjects were monitored using a smartphone and wearables for 6 weeks. Overall, the smartphone application was well tolerated, but 67% of the subjects noticed a reduced battery life on their smartphone. Data completeness was more than 75% for all sensors. FSHD and non-FSHD controls were classified with 93% accuracy, 100% sensitivity, and 80% specificity. Features relating to smartphone acceleration, app usage, location, physical activity, sleep, and call behavior were the most salient features for the classification. The investigators concluded that remote monitoring data collection allowed for the collection of daily activity data, and that it is possible to detect differences in features in FSHD patients and non-FSHD controls using smartphones and wearables based on data related to physical and social activity.

This session was concluded by a presentation by Nicol Voermans (Radboud University Medical Center) on the initiation of the FSHD European Trial Network. The guidelines for clinical trials, pharmaceutical regulation and participation, and health care provisions in European countries differ in various subtle ways and would benefit from an overall strategy specifically catering to the multilingual European situation. This prompted FSHD Europe to launch the FSHD European Trial network, a project in collaboration with the Clinical Research Trial Network, TreatNMD, and the European Reference Networks for Rare Diseases. A virtual kick-off meeting for the network was held in the spring of 2021. This resulted in four working groups on clinical and genetic diagnosis, clinical outcome measures, and biomarkers and muscle imaging. These groups will work toward an application for two ENMC workshops in 2022 and 2023. Furthermore, a communication delegate will be appointed. Network members will be appointed to reach out to countries not yet connected and to collaborate with other organizations, including the FSHD CTRN. Network experts will contribute to the patient expectations survey FSHD Europe is currently performing. The network members will promote the use of the agreed FSHD Core dataset across all clinical centers and patient registries. More information can be found in the following: https://fshd-europe.info/what-is-it-about/.

## Special session—Fulcrum’s phase 2b ReDUX4 results

Rabi Tawil (University of Rochester Medical Center) described the results of ReDUX4, phase 2b trial of losmapimod for the treatment of FSHD sponsored by Fulcrum Therapeutics. ReDUX4 is a randomized, double-blind, placebo-controlled, 48-week study of the efficacy and safety of losmapimod in treating FSHD. Losmapimod is an oral, selective, small-molecule inhibitor of p38α/β MAPK that significantly reduces DUX4 expression in pre-clinical studies. The primary endpoint was change in DUX4-driven gene expression in muscle needle biopsies. Secondary endpoints included safety, pharmacokinetics/dynamics, and change in whole-body musculoskeletal MRI (WB-MSK-MRI). Clinical outcome assessments included changes in reachable workspace (RWS), timed up and go (TUG, FSHD TUG), hand-held dynamometry, motor function measure (MFM), and patient-reported outcome measures (PGIC, FSHD-HI). Eighty subjects with genetically confirmed FSHD1 were randomized 1:1 to losmapimod (15 mg oral BID) or placebo for 48 weeks. No difference was found in DUX4-driven gene expression. At week 48, losmapimod demonstrated significantly slowed progression on muscle fat infiltration (MFI) and significant improvement in relative surface area on RWS with weights. On PGIC, participants reported significant improvement compared to placebo, and assessment of maximum voluntary isometric contraction (MVICT) with hand-held dynamometry showed selective stabilization across several parameters. ReDUX4 has shown evidence of the benefit of treatment with losmapimod on structural (WB-MSK MRI) and FSHD relevant clinical endpoints (PGIC, RWS, and dynamometry). Additionally, losmapimod continued to exhibit favorable safety and tolerability and will be pursued further as a potential disease-modifying treatment for people living with FSHD.

## Best poster prize and young scientist award

The FSHD Society Young Investigator Award this year was presented to Dr. Giorgio Tasca, who is a neurologist at the Fondazione Policlinico Universitario “A. Gemelli” IRCCS in Rome, Italy. Dr. Tasca and his group have published many papers that have significantly advanced our knowledge of the potential of muscle imaging in FSHD as a diagnostic tool, as a biomarker of disease, and as a research tool to investigate disease pathophysiology. His studies have also shed light on the molecular mechanisms involved in the active phases of the disease at a single muscle level and are currently focused on the detection of tissue and circulating biomarkers, which are some of the highest priorities for the FSHD community.

Dr. Alec DeSimone, currently a postdoctoral researcher at Yale School of Medicine, was the winner of the Best Poster Award at the 2021 FSHD IRC. His poster, “A live-cell drug screening platform for FSHD therapeutics” described a cost-effective, higher throughput, and clinically relevant drug screening platform. Using a previously described FSHD patient-derived myoblast cell line with an integrated fluorescent DUX4 reporter [7], DeSimone developed a high content imaging assay to accurately quantify metrics such as number of DUX4 activation events in a myotube and the survival time following DUX4 activation to track DUX4-induced myotoxicity.

## Conclusions and future directions

Advances in the understanding of DUX4 regulation, the consequences of its activation and the pathophysiological mechanisms leading to muscle wasting in FSHD, along with the development of more accurate and multi-systemic disease models, the surge of interventional strategies, as well as the validation of more sensitive outcome measures and the expansion of a trial-ready clinical network reflect a promising landscape for the development of therapies for FSHD. These advances, stemming from basic molecular characterization to multinational clinical coordination, also highlight the many simultaneous facets required to occur in parallel for a field to advance promising therapies. The 2021 FSHD International Research Congress brought together over 350 registered delegates from over 20 countries representing the academic, clinical, regulatory, and industrial sector with expertise and interest in the aforementioned domains. The 2022 FSHD International Research Congress aims to build on this momentum and is being planned for June 16–17 as an in-person event to be held in Orlando, Florida.

### Program committee and co-chairs

Jamshid Arjomand, FSHD Society, USA

Jessica C. de Greef, Leiden University Medical Center, The Netherlands

Davide Gabellini, IRCCS San Raffaele Scientific Institute/Interuniversity Institute of Myology, Italy

Scott Q. Harper, Center for Gene Therapy, The Abigail Wexner Research Institute at Nationwide Children’s Hospital/Department of Pediatrics, The Ohio State University College of Medicine, USA

Lawrence J. Hayward, University of Massachusetts Medical School, USA

Sujatha Jagannathan, University of Colorado Anschutz Medical Campus, USA

June Kinoshita, FSHD Society, USA

Mikell Lang, FSHD Society, USA

Karlien Mul, Radboud University Medical Center, The Netherlands

Sabrina Sacconi, Nice University Hospital/Institute of Research on Cancer and Aging of Nice, France

Mark Stone, FSHD Society, USA

Kyoko Yokomori, School of Medicine, University of California, Irvine, USA

## Data Availability

Not applicable.
